# Long-term methylphenidate use for children and adolescents with attention deficit hyperactivity disorder and risk for depression, conduct disorder, and psychotic disorder: a nationwide longitudinal cohort study in South Korea

**DOI:** 10.1186/s13034-022-00515-5

**Published:** 2022-10-11

**Authors:** Jimyung Park, Dong Yun Lee, Chungsoo Kim, Yo Han Lee, Su-Jin Yang, Sangha Lee, Seong-Ju Kim, Jeewon Lee, Rae Woong Park, Yunmi Shin

**Affiliations:** 1 Department of Biomedical Sciences, Ajou University Graduate School of Medicine, Suwon, South Korea; 2 Department of Biomedical Informatics, Ajou University School of Medicine, Suwon, South Korea; 3 Department of Preventive Medicine, Korea University School of Medicine, Seoul, South Korea; 4 Gwangju Smile Center for Crime Victims, Gwangju, South Korea; 5 Department of Psychiatry, Ajou University School of Medicine, 206, Worldcup-ro, Yeongtong-gu, Suwon, Gyeonggi-do 16499 Republic of Korea; 6 Department of Psychiatry, Soonchunhyang University Bucheon Hospital, Bucheon, South Korea

**Keywords:** Attention-deficit hyperactivity disorder, Methylphenidate, Psychostimulant, Long-term treatment

## Abstract

**Background:**

Methylphenidate (MPH) is the most frequently prescribed medication for the treatment of attention deficit hyperactivity disorder (ADHD). However, the safety of its long-term use remain unclear. In particular, real-world evidence of long-term MPH treatment regarding the risk of depression, conduct disorders, and psychotic disorders in children and adolescents is needed. This study aimed to compare the risks of depression, conduct disorder, and psychotic disorder between long- and short-term MPH treatments in children and adolescents.

**Methods:**

This population-based cohort study used a nationwide claims database of all patients with ADHD in South Korea. Patients aged less than 18 years who were prescribed MPH were included in the study. Long- and short-term MPH were defined as > 1 year, and < 1 year, respectively. Overall, the risk of developing depressive disorder, conduct disorder and oppositional defiant disorder (ODD), and psychotic disorder were investigated. A 1:2 propensity score matching was used to balance the cohorts, and the Cox proportional hazards model was used to evaluate the safety of MPH.

**Results:**

We identified 1309 long-term and 2199 short-term MPH users. Long-term MPH use was associated with a significantly lower risk of depressive (hazard ratio [HR], 0.70 [95% confidence interval [CI] 0.55–0.88]) and conduct disorders and ODD (HR, 0.52 [95% CI 0.38–0.73]) than short-term MPH use. Psychotic disorder was not significantly associated with long-term MPH use (hazard ratio [HR], 0.83 [95% confidence interval [CI] 0.52–1.32]).

**Conclusions:**

Our findings suggest that long-term MPH use may be associated with a decreased risk of depression, conduct disorders and ODD. Moreover, the long-term use of MPH does not increase the risk of psychotic disorders. Long-term MPH administration may be considered as a favourable treatment strategy for children and adolescents with ADHD regarding depressive, conduct, and psychotic disorders.

**Supplementary Information:**

The online version contains supplementary material available at 10.1186/s13034-022-00515-5.

## Background

Attention deficit hyperactivity disorder (ADHD) is a common neurodevelopmental disorder that occurs in childhood and adolescence. ADHD is a chronic debilitating disorder that can affect several aspects of an individual’s life including academic performance, peer relationships, and parent–child relationships [1]. Several studies have suggested that 40–60% of affected children continue to show symptoms of the disorder until adulthood [2, 3]. Children and adolescents with ADHD are at an increased risk of comorbidities, including conduct problems, mood and anxiety disorders, and substance abuse [4]. Moreover, patients with ADHD and comorbidities are at an increased risk of adverse outcomes. Youths with ADHD and depression had a greater risk of suicide [5]. Adolescents with ADHD and conduct disorders are more likely to experience substance abuse [6]. Therefore, drug treatment is required to mitigate symptoms and related impairments of ADHD. The American Academy of Pediatrics and American Academy of Child and Adolescent Psychiatry guidelines recommend medication as the first-line treatment [7, 8].

Methylphenidate (MPH) is the most frequently used medication for ADHD treatment. As the prescription of MPH for both children and adults has increased in several countries [9–11], concerns have been raised regarding the safety of its long-term use [12]. In fact, approximately one-third of patients with ADHD (both children and adults) continue to take MPH for 2 years after treatment initiation [13]. The Committee for Medicinal Products for Human Use has suggested that more studies are needed on the long-term effects of MPH, especially on adverse psychiatric events [14]. A recent review has investigated the association between long-term MPH treatment and adverse neuropsychiatric effects [15]. However, it was shown that the evidence was unclear, and more data are needed on the relationship between long-term MPH use and adverse neuropsychiatric effects. Although the Comparison of Methylphenidate and Psychotherapy in the Adult ADHD Study (COMPAS) was conducted focusing on safety profiles including neuropsychiatric events, it only included adult patients with ADHD [16]. Therefore, research is required to establish real-world evidence for the impact of long-term MPH treatment on adverse neuropsychiatric events in children and adolescents with ADHD.

In this study, we targeted depressive disorders and conduct disorders, which are the most common comorbidities of ADHD, and psychotic disorders, where the risk of MPH use was reported [17, 18]. We aimed to evaluate the risk of depressive disorders, conduct disorders, and psychotic disorders associated with long-term MPH treatment compared with short-term MPH treatment in children and adolescent patients with ADHD.

## Methods

### Data source

This observational cohort study used data from a nationwide claims database in South Korea. The database was obtained from the Health Insurance Review and Assessment Service (HIRA), a national institution for national health insurance that covers the entire South Korean population. HIRA claims data are generated when healthcare service providers submit a claim to be reimbursed for a service provided [19]. HIRA claims data consist of demographics, diagnosis (using International Classification of Disease (ICD) codes), procedures, prescription drug information (using Anatomical Therapeutic Chemical (ATC) codes), medical material, and healthcare resources [20]. Prescription drug information was based on the pharmacy records of dispensed prescriptions. In this study, we obtained data from all patients with ADHD (n = 336,098) in South Korea who were enrolled in the national health insurance scheme from January 2016 to March 2021. The cohort was converted into the Observational Medical Outcomes Partnership–Common Data Model (OMOP–CDM) version 5 format, an anonymised and standardised medical database format proposed by the Observational Health Data Sciences and Informatics (OHDSI) consortium [21]. This study was approved by the Institutional Review Board of the Ajou University Hospital (IRB number: AJIRB-MED-EXP-21-088), which waved the requirement for informed consent.

### Study population and exposure

**Fig. 1:**
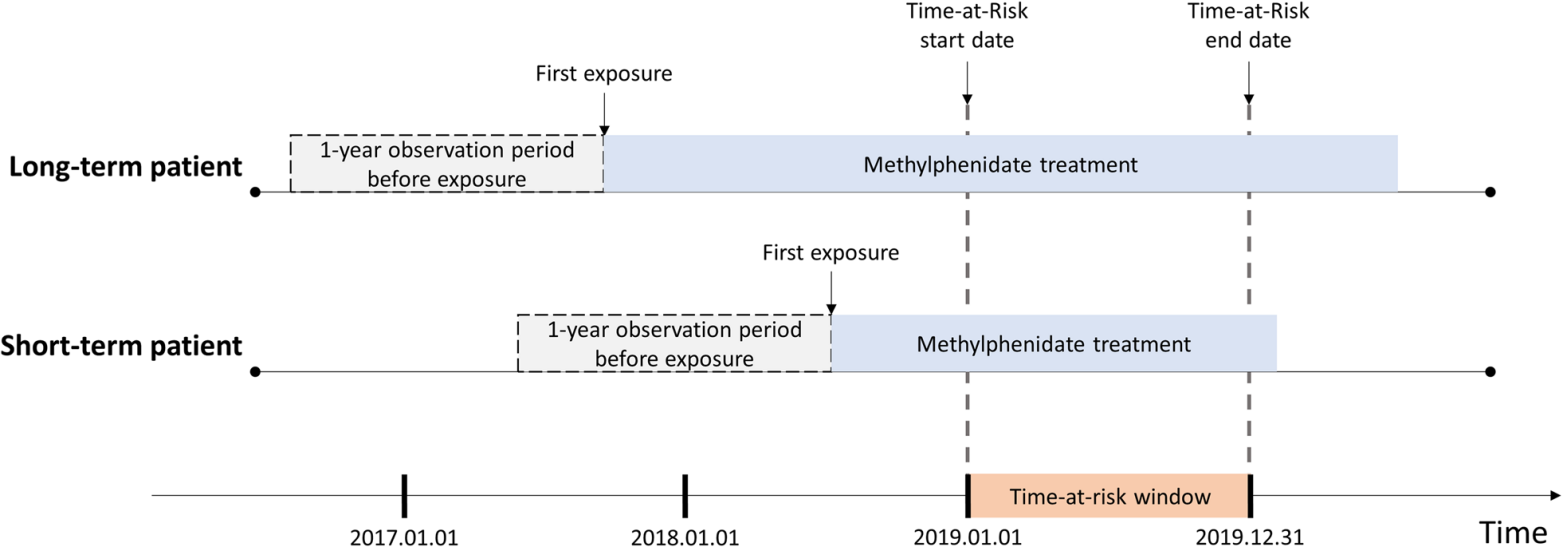
Schema of time-at-risk windows of long-term and short-term methylphenidate users

We defined the ADHD cohort as patients aged between 6 and 17 years who were diagnosed with ADHD (ICD 10th edition: F90.0, F90.1, F90.2, F90.8, and F90.9). The index date was defined as 1 January 2019. Considering that 2019 was the time-at-risk window, the patients were required to have continuous MPH treatment from 1 January 2019 to 31 December 2019 to assess the risk of the outcomes during MPH treatment (Fig. 1). To verify their first exposure to MPH treatment, we excluded patients who had been enrolled in the database for less than 1 year before MPH treatment. Patients were divided into two groups according to the time of MPH initiation: long-term and short-term MPH treatment groups [22, 23]. Long-term MPH treatment was defined as continuous MPH exposure since 2017, indicating an MPH exposure of at least 365 days and less than 730 days. Short-term MPH treatment was defined as continuous MPH exposure since 2018, indicating an MPH exposure of less than 365 days. That is, the defined treatment period was a 12-month cutoff. MPH prescription within 30 days after prior MPH prescription was considered a continuous medication treatment.

### Outcomes

All outcomes were defined based on their diagnostic codes according to the SNOMED-CT classification (Additional file 1: Table S1) and included only the first diagnosed events. The outcomes consisted of depressive disorder, psychotic disorder, and conduct disorder and oppositional defiant disorder (ODD). Three outcomes were included in this study.

### Statistical analysis

In this study, the time-at-risk window started on 1 January 2019 to simultaneously compare the effects of MPH treatment duration between long- and short-term MPH users (Fig. 1). Patients were followed up until 31 December 2019, which was the last date of the time-at-risk window.

Propensity score matching was used to balance the baseline characteristics between long- and short-term MPH users [24]. To reduce patient exclusion, a 1:2 nearest neighbour matching of patients with the same propensity scores was used. The absolute standardised mean difference (aSMD) was used to describe the balance of the covariate distribution. Patient demographics (age and sex), daily MPH dose, and neuropsychiatric disorders (anxiety disorder, bipolar disorder, autism spectrum disorder, sleep disorder, and tic disorder) were used to match covariates. Additionally, we matched the outcomes of interest (depression, psychotic disorder, conduct disorder and ODD) to reduce bias. For example, if depression was the target outcome, psychotic disorder and conduct disorder and ODD were balanced between the two groups. To estimate hazard ratios (HRs) between MPH treatment duration and outcomes, we used Cox proportional hazard regression models. Statistical significance was set at *P* < 0.05.

### Sensitivity analysis

To assess the robustness of the findings, three sensitivity analyses were conducted using different definitions of treatment periods, exclusion of non-stimulant ADHD medications, and comparison to non-MPH users. First, in addition to defined treatment periods using a 12-month cutoff, 2 additional treatment periods were performed: (1) 9-month cutoff; and (2) 15-month cutoff. Second, we conducted sensitivity analysis regarding non-stimulant ADHD medications. The primary analysis included patients taking non-stimulant ADHD medications (atomoxetine, bupropion, and clonidine). In the sensitivity analysis, patients who were prescribed non-stimulant ADHD medications were excluded from the comparison of the pure effects of MPH treatment duration. Third, to further clarify the association between MPH and outcomes, we compared ADHD patients by dividing them into MPH users and non-MPH users. Specifically, we performed comparisons between long-term MPH users and non-MPH users, and between short-term MPH users and non-MPH users. Non-MPH users were defined as patients newly diagnosed with ADHD since 2017 and not using MPH during follow-up. In this sensitivity analysis, the defined treatment period between long-term and short-term was a 12-month cutoff.

## Results

### Baseline characteristics

**Fig. 2:**
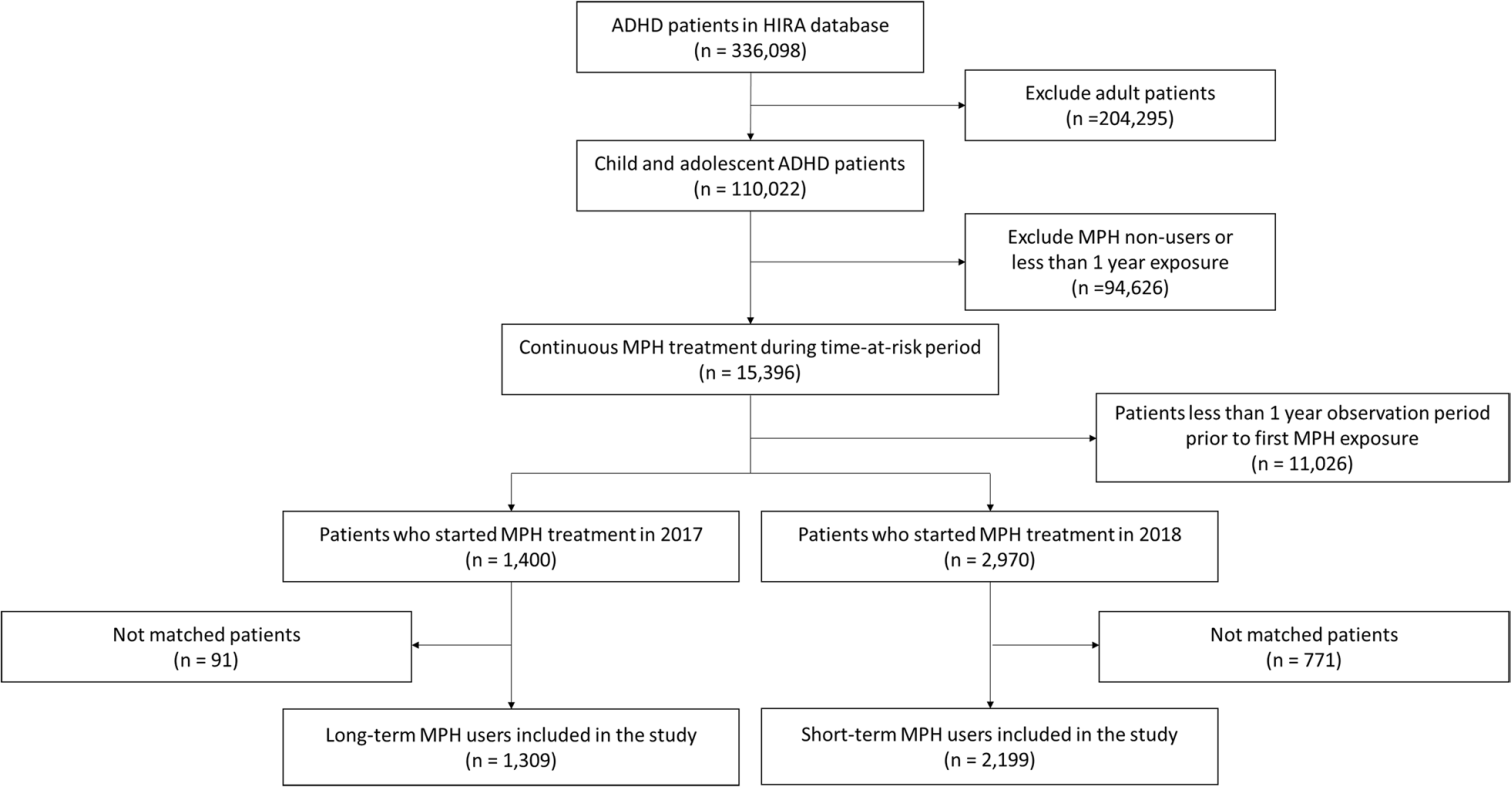
Flowchart of the study population investigating the incidence of depressive disorder

Regarding the outcomes of depressive disorder, a total of 3508 patients receiving MPH treatment from the National Health Claims database were included in the study: 1309 long-term MPH users and 2199 short-term MPH users (Fig. 2). The baseline characteristics of the primary analysis regarding the outcomes of depressive disorders are described in Table 1. The baseline characteristics and flow chart regarding other outcomes are shown in (Additional file 1: Table S2, S3 and Fig S1, S2). The mean daily MPH dose for the long- and short-term MPH users after propensity score matching were 22.2 $\pm$ 8.4 mg and 20.9 $\pm$ 8.2 mg, respectively. Additionally, the mean age of long-term and short-term users were 8.8 $\pm$ 2.6 years and 8.6 $\pm$ 2.4 years. After propensity score matching, all baseline characteristics between the long-term and short-term MPH groups were balanced according to outcomes (all aSMD < 0.20; Table 1 and Additional file 1: Fig S3). Additionally, all baseline characteristics in the all settings of sensitivity analysis were balanced according to the outcomes (all aSMD < 0.20; Additional file 1: Fig S4). Overall, the baseline characteristics of the two groups showed no significant differences in any of the covariates.

**Table 1:** Baseline characteristics of the study population investigating depressive disorder in the primary analysis

	Before matching			After matching		
	Long-term users (n = 1400)	Short-term users (n = 2970)	Absolute SMD	Long-term users (n = 1309)	Short-term users (n = 2199)	Absolute SMD
Age, mean (SD)	8.6 $\pm$ 2.4	8.8 $\pm$ 2.6	0.08	8.6 $\pm$ 2.4	8.6 $\pm$ 2.4	0.01
Gender, male	1209 (86.4)	2437 (82.1)	0.13	1132 (85.8)	1866 (84.8)	0.01
Neuropsychiatric disease history, n (%)						
Anxiety disorder	118 (8.4)	206 (6.9)	0.14	99 (7.5)	170 (7.7)	0.05
Bipolar disorder	107 (7.6)	134 (4.5)	0.14	82 (6.2)	116 (5.3)	0.02
Autism spectrum disorder	109 (7.8)	197 (6.6)	0.14	89 (6.7)	169 (7.7)	0.02
Sleep disorder	17 (1.2)	33 (1.1)	0.03	16 (1.2)	26 (1.2)	0.01
Tic disorder	152 (10.9)	182 (6.1)	0.16	128 (9.7)	167 (7.6)	0.03
Conduct disorder and ODD	162 (11.6)	262 (8.8)	0.18	129 (9.8)	224 (10.2)	0.05
Psychotic disorder	59 (4.2)	43 (1.4)	0.12	55 (4.2)	38 (1.7)	0.05
Daily methylphenidate dose, mean (SD)	22.5 $\pm$ 8.6	19.1 $\pm$ 8.4	0.39	22.2 $\pm$ 8.4	20.9 $\pm$ 8.2	0.02

*SMD* Standardised mean difference

### Primary analysis

**Fig. 3:**
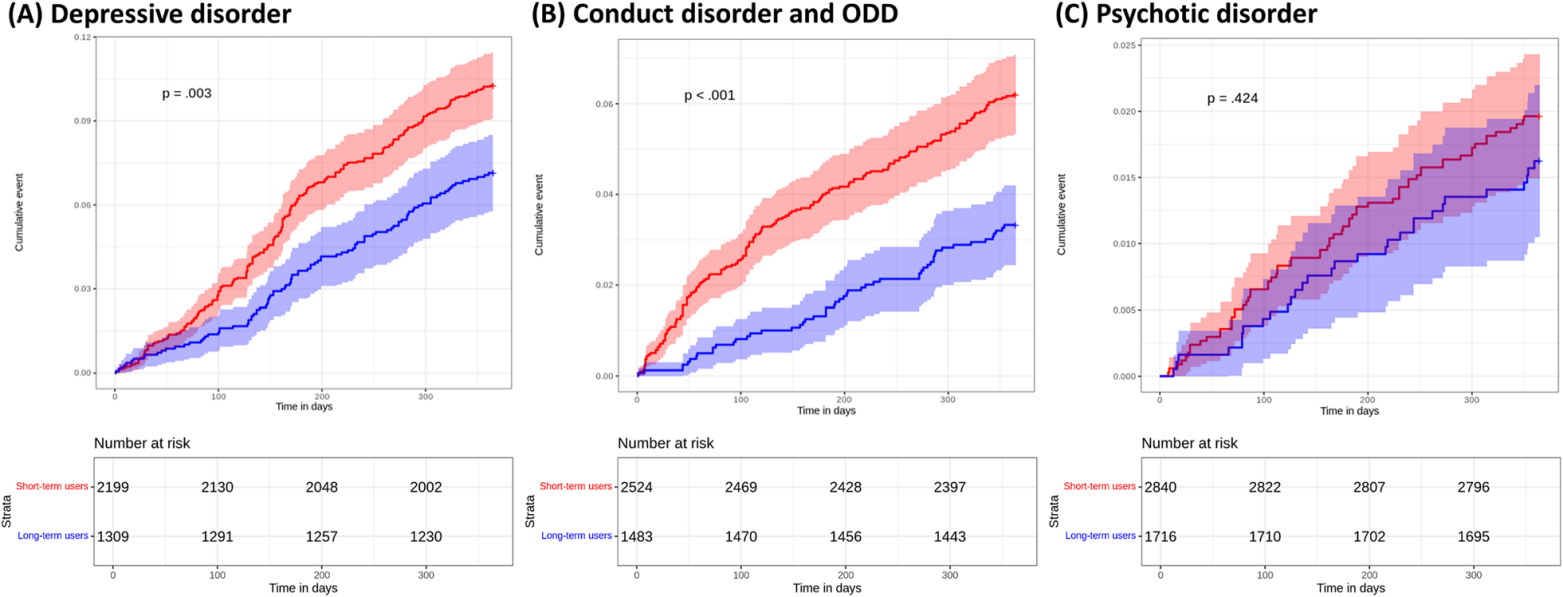
Cumulative incidence plots investigating outcomes of interest in the primary analysis

**Fig. 4:**
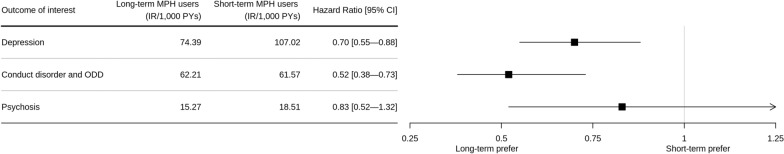
Cox’s proportional hazards model for depressive disorder, conduct disorder, and psychotic disorder stratified by duration of MPH usage in ADHD youths

The survival curves for the primary analysis after propensity score matching are presented in Fig. 3. In the primary analysis, the risk of depressive disorder was statistically significantly lower in the long-term MPH use than short-term MPH use (HR, 0.70 [95% CI 0.55–0.88]; *P* = 0.003) (Fig. 4). In addition to depressive disorder, the risk of conduct disorder and ODD was statistically significantly lower in the long-term MPH use than short-term MPH use (HR, 0.52 [95% CI 0.38–0.73]; *P* < 0.001). However, the risk of psychotic disorder was not statistically significantly different between long- and short-term MPH use in the primary analysis (HR, 0.83 [95% CI, 0.52–1.32]; *P* = 0.424).

### Sensitivity analysis

Sensitivity analyses regarding different treatment periods are presented in Additional file 1 Table S4. In a 9-month cutoff, the risk of depressive disorder and conduct disorder and ODD was statistically significantly lower in the long-term MPH use than short-term MPH use (depressive disorder: HR, 0.56 [95% CI 0.43–0.74]; *P* < 0.001; conduct disorder and ODD: HR, 0.66 [95% CI 0.47–0.91]; *P* = 0.012, respectively). The risk of psychotic disorder was not statistically significantly different between long- and short-term MPH use (HR, 0.60 [95% CI 0.31–1.13]; *P* = 0.112). In a 15-month cutoff, the risk of depressive disorder and conduct disorder and ODD was statistically significantly lower in the long-term MPH use than short-term MPH use (depressive disorder: HR, 0.73 [95% CI 0.57–0.93]; *P* = 0.010; conduct disorder and ODD: HR, 0.55 [95% CI 0.39–0.77]; *P* < 0.001, respectively). The risk of psychotic disorder was not statistically significantly different between long- and short-term MPH use (HR, 0.95 [95% CI 0.59–1.52]; *P* = 0.829).

In the sensitivity analysis excluding patients with non-stimulant ADHD medications, consistent result with primary analysis was observed (Additional file 1: Table S5). Long-term MPH use was statistically significantly associated with a lower risk of depressive disorder (HR, 0.69 [95% CI 0.52–0.91]; *P* = 0.009) and conduct disorder and ODD (HR, 0.43 [95% CI 0.28–0.67]; *P* < 0.001) than short-term MPH use. The risk of psychotic disorder was not statistically significantly different between two groups (HR, 1.21 [95% CI 0.65–2.25]; *P* = 0.548).

The sensitivity analysis comparing MPH users and non-MPH users in ADHD patients is presented in (Additional file 1: Table S6). Long-term MPH use was statistically significantly associated with a lower risk of depressive disorder (HR, 0.59 [95% CI 0.47–0.74]; *P* < 0.001) and conduct disorder and ODD (HR, 0.58 [95% CI 0.43–0.79]; *P* < 0.001) than non-MPH use. The risk of psychotic disorder was not statistically significantly different between two groups (HR, 0.82 [95% CI 0.54–1.24]; *P* = 0.348). However, the risk of depressive disorder, conduct disorder and ODD, and psychotic disorder was not statistically significantly different between short-term MPH use and non-MPH use (depressive disorder: HR, 0.91 [95% CI 0.79–1.04]; *P* = 0.157; conduct disorder and ODD: HR, 1.09 [95% CI 0.91–1.29]; *P* = 0.349; psychotic disorder: HR, 0.88 [95% CI 0.66–1.19]; *P* = 0.406, respectively).

## Discussion

In this nationwide cohort study, we found that long-term MPH treatment for children and adolescents with attention deficit hyperactivity disorder was associated with a significantly lower risk of depressive and conduct disorders than short-term MPH treatment. No significant differences were noted in the risks of psychotic disorders between the long- and short-term MPH treatment groups. These results were consistent across the analysis using different treatment periods and in the analysis excluding non-stimulant medication. In addition, compared to the non-MPH users, consistent findings regarding long-term MPH users were observed. However, the risk of depressive disorder, conduct disorder and ODD, and psychotic disorder was not significantly different between short-term MPH users and non-MPH users.

Although it is a necessary to investigate the long-term treatment effects of MPH, the current evidence is limited [25]. Depression is known to be the most common comorbidity of ADHD. Depression in children and adolescents with ADHD usually occurs after its onset. Longitudinal studies have shown that MPH treatment may reduce the risk of subsequent depression in adolescents with ADHD [26, 27]. Population-based pharmacoepidemiological studies have found that ADHD medication is associated with the reduced probability of developing depression [28, 29]. Specifically, the patterns observed in our study align with those of Chang et al. showing that the risk of depression was lower for longer duration of ADHD medication [29]. Levels of depression improved during the first year of treatment compared to the levels after a brief MPH treatment. Improvements in academic and social functional domains by long-term stimulant treatment and the resulting alternative development trajectory may be the reason for the reduction in depression [27, 30]. Additionally, long-term MPH treatment was associated with a lower risk of conduct disorder and ODD than short-term MPH treatment. Our results are consistent with previous findings that stimulant treatment reduces the risk of developing conduct disorder in both boys and girls with ADHD [31]. A recent qualitative review of studies examining the effects of ADHD medications has shown a reduced relative risk of injuries, motor vehicle accidents, and substance abuse [32, 33]. It have been reported to have a therapeutic effect of psychostimulants on the management of oppositional behaviour, conduct problems, and aggression in ADHD patients without ODD or CD [34]. It is possible that ADHD medication helps patients to organise their lives better and it contributes to continuing changes at the neuronal level [35]. One of the most common comorbidities associated with ADHD is conduct disorder and/or ODD. Conduct disorder and/or ODD are present in approximately 40–70% of children with ADHD [36]. The symptoms of both disorders usually respond to psychostimulants; however, ADHD with conduct disorder usually has a worse clinical outcome than either of the two conditions alone [37]. Given the poor prognosis associated with conduct disorder and ODD, the effects of stimulants are likely to have beneficial effects in people with ADHD. Dopaminergic excess induced by MPH treatment may trigger psychotic symptoms [38]. Although a previous study showed that MPH treatment led to an increased risk of psychotic disorders, this study did not distinguish between this risk and long- and short-term MPH treatments [39]. Also, a study using Hong Kong population-based electronic medical records reported that risk of psychotic disorders was not found during MPH exposure compared to non-exposure [18]. A possible explanation for the increased risk of psychotic disorders is that ADHD itself is a risk factor for psychosis [39]. In our study, no difference was noted in the risk of psychotic disorder between the long- and short-term MPH treatment groups.

Compared to non-MPH users, long-term MPH users were associated with a significantly lower risk of depressive and conduct disorders. The risk of psychotic disorder was not significantly different between two groups. However, no significant differences were noted in the risks of all outcomes between the short-term MPH users and non-MPH users. Although previous studies suggested beneficial effects of MPH on neuropsychiatric outcomes [25], this study found that the effects of MPH use on depression and conduct disorders were different depending on the duration of MPH use. It is possible that maintaining MPH for more than a certain period may have beneficial effects on depression and conduct disorders. Considering the risk of depression and conduct disorders was significantly lower in the long-term MPH use than in the short-term MPH use in the primary analysis, these results were consistent with the findings of the primary analysis.

This was a population-based cohort study. Although randomised controlled trials (RCTs) are the gold standard for the evaluation of health care outcomes, the cohort’s strengths, such as longer follow-up time and larger sample size, could be an alternative to RCTs [25]. To overcome potential confounding factors in this population-based study, we applied the propensity score matching to the study population. The propensity score was used to reduce the effects of confounding factors [40]. Specifically, a 1:2 nearest neighbour matching for patients with the same propensity scores was used, considering the ratio between the two groups. And we conducted sensitivity analyses excluding patients who were treated with non-stimulant ADHD medications. Meanwhile, we compared the risks of depressive, conduct, and psychotic disorders according to the duration of MPH use. Several previous studies have compared the effects of the treatment duration of MPH. Huang et al. and Liang et al. divided patients with ADHD into long and short-term users according to the cumulative defined daily dose [41, 42]. Schrantee et al. classified patients with ADHD according to the time of drug initiation [43]. Also, several studies classified short-term and long-term based on one year [22, 23, 40]. Likewise, we compared ADHD patients by dividing them into short-term and long-term groups based on one year.

The strengths of this study include the use of national health insurance data that contained data from all patients with ADHD in South Korea. Furthermore, we evaluated the risk of common comorbidities of ADHD according to the length of MPH use. To increase comparability, we matched the doses that may have caused side effects. Considering the sex differences in ADHD symptoms, we also matched the sex ratio between the two groups.

This study has several limitations. First, we were unable to distinguish between the types of ADHD in patients. This was because it was difficult to identify the detailed symptoms owing to the nature of the claims data. Second, we could not include familial factors related to ADHD because of the limitations of the claim database. Given strong genetic background of ADHD, further studies that include familial factors are needed. Third, one year may not be accurate time point to distinguish between short-term and long-term MPH use. In fact, the relationships between 11 and 13 months of use may be closer to that of between 11 and 4 months. However, consistent findings were demonstrated across different treatment periods. Fourth, there was no information regarding the patients’ management except for the number of prescription days. It may not be possible to determine the level of treatment compliance or the effect on the parents of the child.

## Conclusions

Our findings suggest a decreased risk of depression and conduct disorders in patients undergoing long-term MPH treatment. No difference in the risk of psychotic disorders was shown between the short- and long-term MPH treatments. These findings support the notion that long-term MPH treatment may not be contraindicated for depression, conduct disorders, and psychotic disorders in children and adolescents with ADHD. There is a possibility of unmeasured confounders; hence, further research is required to clarify the safety of long-term MPH use.

## Supplementary Information

**Additional file 1: Figure S1.** Flowchart of the study population investigating the incidence of conduct disorder and ODD. **Figure S2.** Flowchart of the study population investigating the incidence of psychotic disorder. **Figure S3.** Absolute standardized mean differences before and after propensity score matching in the primary analysis. A, standardized mean differences when outcome is depressive disorder. B, standardized mean differences when outcome is conduct disorder and ODD. C, standardized mean differences when outcome is psychotic disorder. **Figure S4.** Absolute standardized mean differences before and after propensity score matching in the all settings of sensitivity analysis. **Table S1.** Standardized OMOP code list used in the cohort definition. **Table S2**. Baseline characteristics of the study population investigating conduct disorder and ODD in the primary analysis. **Table S3.** Baseline characteristics of the study population investigating psychotic disorder in the primary analysis. **Table S4.** Comparison between methylphenidate long-term and short-term users among ADHD patients using different treatment periods. **Table S5.** Comparison between methylphenidate long-term and short-term users among ADHD patients who never exposed to other anti-ADHD medications. **Table S6.** Comparison between methylphenidate non-users and methylphenidate long-term/short-term users.

## Data Availability

The data that support the findings of this study are available from Health Insurance Review and Assessment Service (HIRA) but restrictions apply to the availability of these data, which were used under license for the current study, and so are not publicly available. Data are however available from the authors upon reasonable request and with permission of HIRA.

## Abbreviations

ADHD: Attention Deficit Hyperactivity Disorder
aSMD: Absolute Standardised Mean Difference
ATC: Anatomical Therapeutic Chemical
CI: Confidence Interval
COMPAS: Comparison of Methylphenidate and Psychotherapy in the Adult ADHD Study
HIRA: Health Insurance Review and Assessment Service
HR: Hazard Ratio
ICD: International Classification of Disease
MPH: Methylphenidate
ODD: Oppositional Defiant Disorder
OHDSI: Observational Health Data Sciences and Informatics
OMOP–CDM: Observational Medical Outcomes Partnership–Common Data Model
