# Supplementary material for: Long-term methylphenidate use for children and adolescents with attention deficit hyperactivity disorder and risk for depression, conduct disorder, and psychotic disorder: a nationwide longitudinal cohort study in South Korea
Source: Child Adolesc Psychiatry Ment Health. 2022 Oct 11;16:80. doi: 10.1186/s13034-022-00515-5 (PMC9554986; doi:10.1186/s13034-022-00515-5)
Supplement: Supplementary file 1 — Additional file 1: Figure S1. Flowchart of the study population investigating the incidence of conduct disorder and ODD. Figure S2. Flowchart of the study population investigating the incidence of psychotic disorder. Figure S3. Absolute standardized mean differences before and after propensity score matching in the primary analysis. A, standardized mean differences when outcome is depressive disorder. B, standardized mean differences when outcome is conduct disorder and ODD. C, standardized mean differences when outcome is psychotic disorder. Figure S4. Absolute standardized mean differences before and after propensity score matching in the all settings of sensitivity analysis. Table S1. Standardized OMOP code list used in the cohort definition. Table S2. Baseline characteristics of the study population investigating conduct disorder and ODD in the primary analysis. Table S3. Baseline characteristics of the study population investigating psychotic disorder in the primary analysis. Table S4. Comparison between methylphenidate long-term and short-term users among ADHD patients using different treatment periods. Table S5. Comparison between methylphenidate long-term and short-term users among ADHD patients who never exposed to other anti-ADHD medications. Table S6. Comparison between methylphenidate non-users and methylphenidate long-term/short-term users. [file 13034_2022_515_MOESM1_ESM.docx]

**Additional file 1**

[Figure S1. Flowchart of the study population investigating the incidence of conduct disorder and ODD 2](#_Toc114671023)

[Figure S2. Flowchart of the study population investigating the incidence of psychotic disorder 3](#_Toc114671024)

[Figure S3. Absolute standardized mean differences before and after propensity score matching in the primary analysis. A, standardized mean differences when outcome is depressive disorder. B, standardized mean differences when outcome is conduct disorder and ODD. C, standardized mean differences when outcome is psychotic disorder. 4](#_Toc114671025)

[Figure S4. Absolute standardized mean differences before and after propensity score matching in the all settings of sensitivity analysis. 5](#_Toc114671026)

[Table S2. Baseline characteristics of the study population investigating conduct disorder and ODD in the primary analysis 17](#_Toc114671027)

[Table S3. Baseline characteristics of the study population investigating psychotic disorder in the primary analysis 18](#_Toc114671028)

[Table S4. Comparison between methylphenidate long-term and short-term users among ADHD patients using different treatment periods 19](#_Toc114671029)

[Table S5. Comparison between methylphenidate long-term and short-term users among ADHD patients who never exposed to other anti-ADHD medications 20](#_Toc114671030)

[Table S6. Comparison between methylphenidate non-users and methylphenidate long-term/short-term users 21](#_Toc114671031)

# Figure S1. Flowchart of the study population investigating the incidence of conduct disorder and ODD


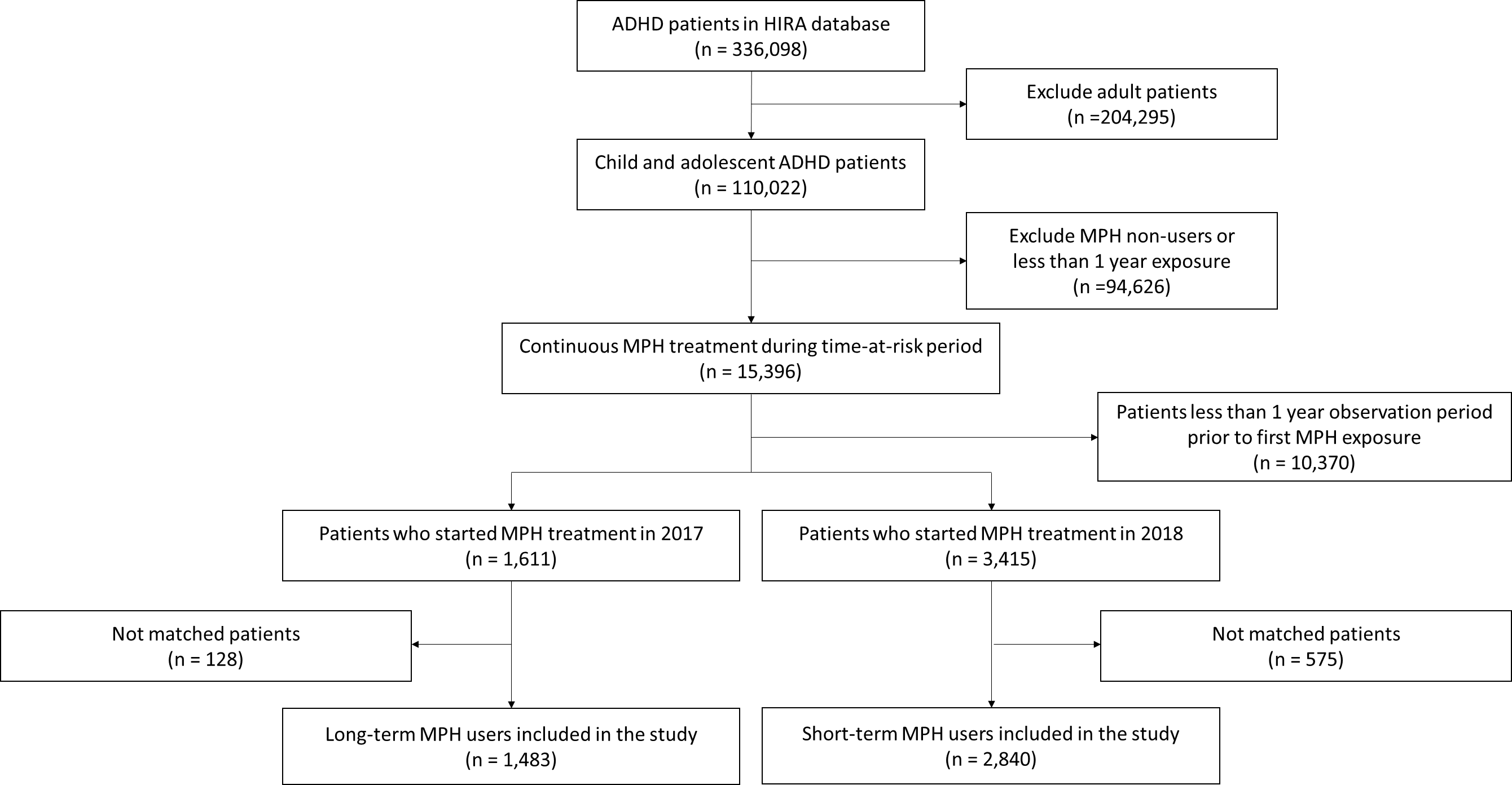


# Figure S2. Flowchart of the study population investigating the incidence of psychotic disorder


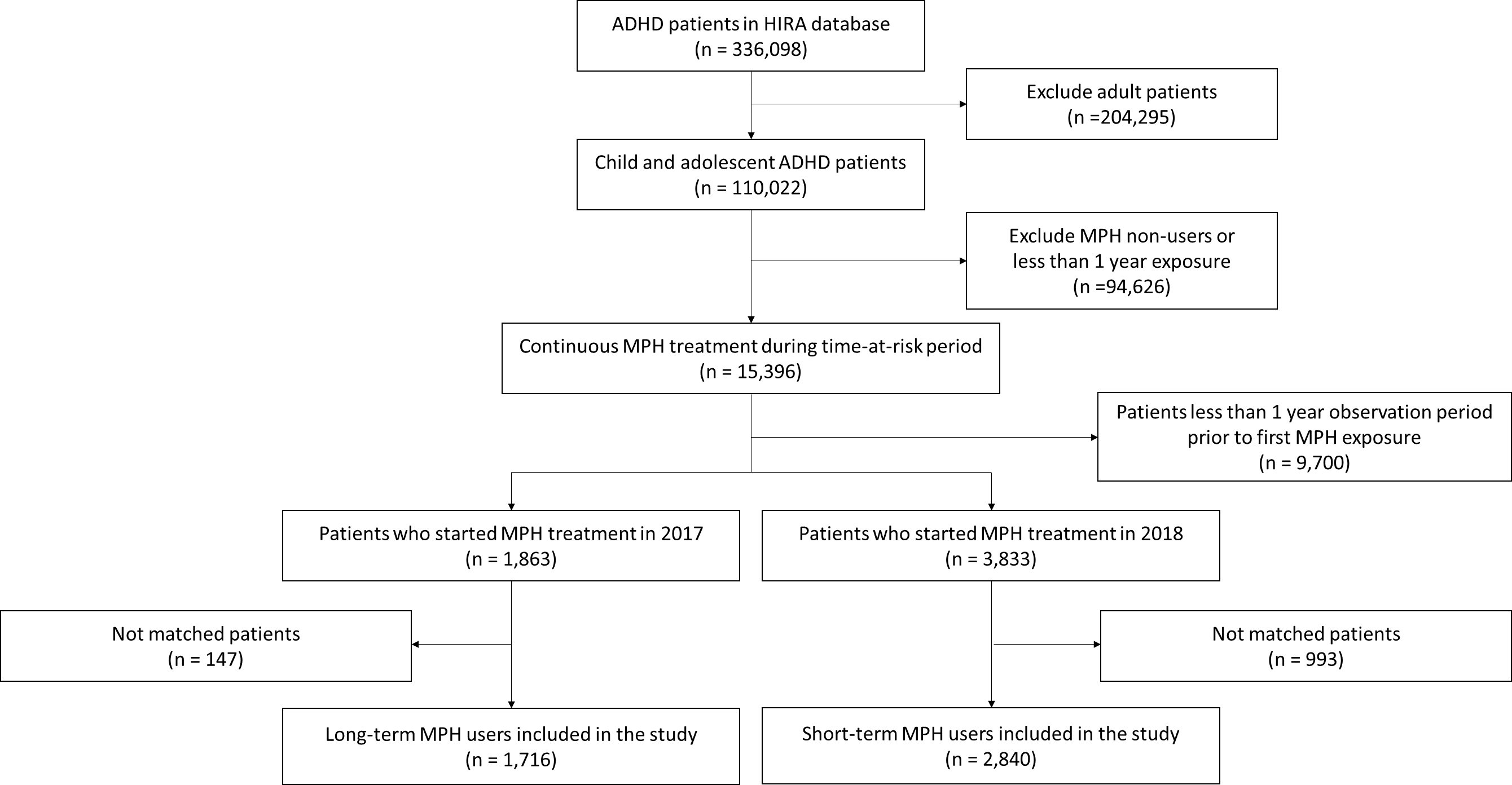


# Figure S3. Absolute standardized mean differences before and after propensity score matching in the primary analysis. A, standardized mean differences when outcome is depressive disorder. B, standardized mean differences when outcome is conduct disorder and ODD. C, standardized mean differences when outcome is psychotic disorder.


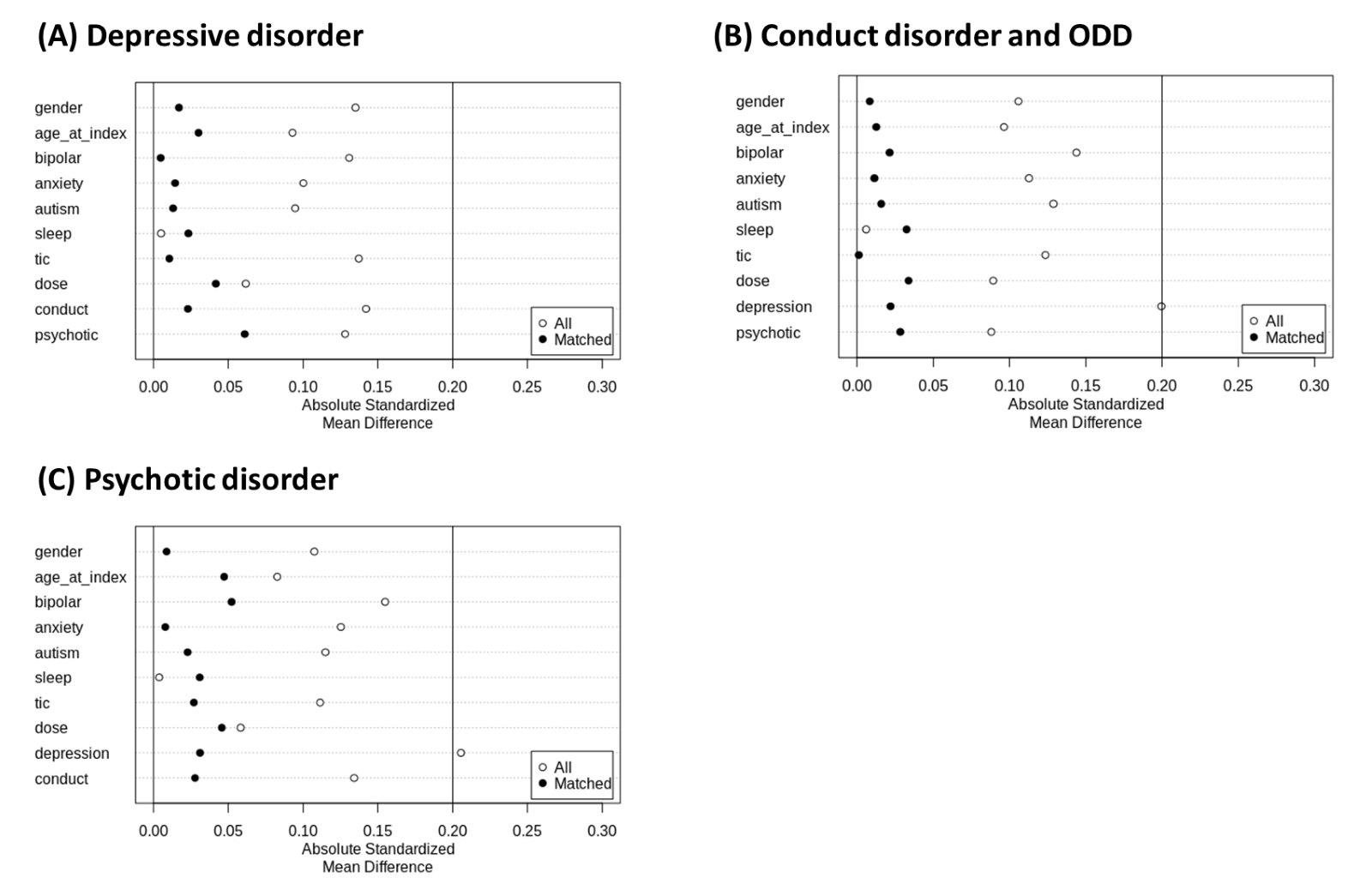


# Figure S4. Absolute standardized mean differences before and after propensity score matching in the all settings of sensitivity analysis.


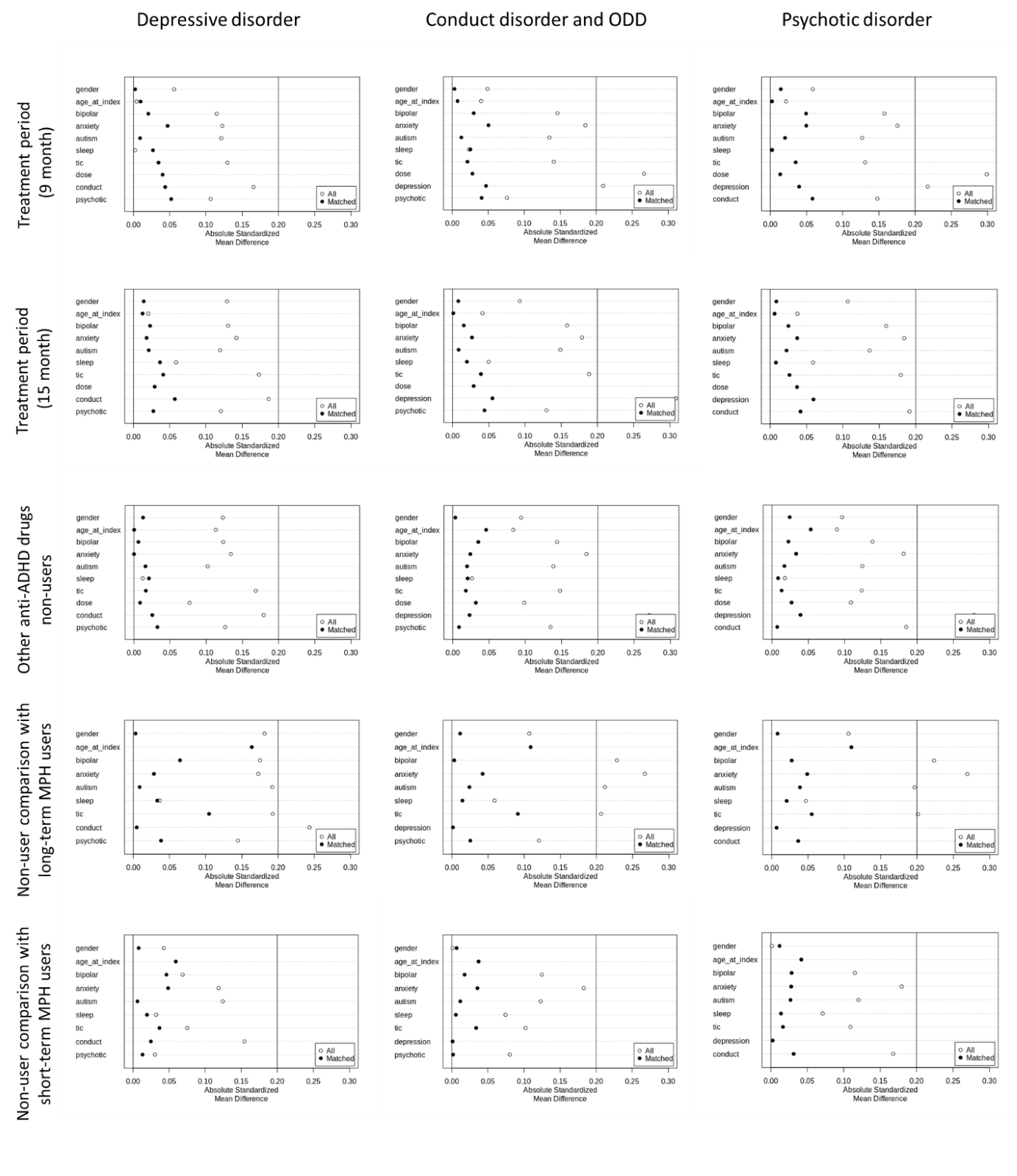


Table S1. Standardized OMOP code list used in the cohort definition

| **Variables** | **Vocabulary** | **OMOP Vocabulary Codes** |
| --- | --- | --- |
| Attention-deficit/hyperactivity disorder | SNOMED | 45765796 (Attention deficit hyperactivity disorder, inattentive presentation),  44784525 (Attention deficit hyperactivity disorder, predominantly hyperactive impulsive type in remission),  44782517 (Attention deficit hyperactivity disorder, predominantly inattentive type in remission),  4253962 (Attention deficit hyperactivity disorder, predominantly hyperactive impulsive type),  4149904 (Attention deficit hyperactivity disorder, combined type),  4149353 (Attention deficit hyperactivity disorder, predominantly inattentive type),  4049391 (Undifferentiated attention deficit disorder),  4047120 (Disorders of attention and motor control),  4041692 (Deficits in attention motor control and perception),  440086 (Child attention deficit disorder),  438409 (Attention deficit hyperactivity disorder),  438132 (Hyperkinetic conduct disorder),  437261 (Hyperkinesis with developmental delay) |
| Depressive disorder | SNOMED | 45757213 (Depressive disorder in mother complicating childbirth)  45757196 (Major depressive disorder in mother complicating pregnancy)  45757195 (Major depressive disorder in mother complicating childbirth)  44813499 (Recurrent major depressive episodes, in remission)  44806193 (Antenatal depression)  44805669 ([X]Recurrent major depressive episodes, severe, with psychosis, psychosis in remission)  44805668 ([X]Single major depressive episode, severe, with psychosis, psychosis in remission)  44805550 (Single major depressive episode, in remission)  44805549 (Recurrent major depressive episodes, in partial remission)  44805542 (Recurrent major depressive episodes, severe)  44782943 (Depressive disorder in remission)  44782720 (Severe seasonal affective disorder)  44782518 (Adjustment disorder with depressed mood in remission)  43531624 (Severe recurrent major depression)  43020483 (Reactive depressive psychosis, single episode)  42872722 (Severe major depression)  42872411 (Severe major depression, single episode)  42538736 (Mood disorder with depressive symptoms caused by synthetic cathinone)  42538604 (Mood disorder with depressive symptoms caused by dissociative drug)  42538601 (Mood disorder with depressive symptoms caused by ecstasy type drug)  42538599 (Mood disorder with depressive symptoms caused by volatile inhalant)  42538596 (Mood disorder with depressive symptoms caused by hallucinogen)  42538590 (Mood disorder with depressive symptoms caused by stimulant)  42538584 (Mood disorder with depressive symptoms caused by opioid)  42534817 (Postpartum major depression in remission)  40481798 (Chronic depressive personality disorder)  37312479 (Antenatal depression)  37309680 (Depressive disorder caused by methamphetamine)  37209503 (Depressive disorder caused by amphetamine)  37117211 (Mood disorder with depressive symptoms caused by sedative)  37111697 (Major depression with psychotic features)  37110438 (Mood disorder with depressive symptoms caused by cocaine)  37110429 (Mood disorder with depressive symptoms caused by anxiolytic)  37110428 (Mood disorder with depressive symptoms caused by hypnotic)  37109952 (Mood disorder with mixed manic and depressive symptoms caused by alcohol)  37109950 (Mood disorder with depressive symptoms caused by alcohol)  37109054 (Severe major depressive disorder co-occurrent with anxiety single episode)  37109053 (Moderate major depressive disorder co-occurrent with anxiety single episode)  37109052 (Mild major depressive disorder co-occurrent with anxiety single episode)  37018656 (Depressive disorder in mother complicating pregnancy)  37016718 (Acute depression)  36717389 (Moderately severe major depression single episode)  36717092 (Moderately severe depression)  36715000 (Minimal major depression)  36714999 (Minimal major depression single episode)  36714998 (Moderately severe recurrent major depression)  36714997 (Minimal recurrent major depression)  36714389 (Moderately severe major depression)  36713698 (Minimal depression)  36712668 (Perinatal depression)  36684319 (Adjustment disorder with mixed anxiety and depressed mood)  35615155 (Recurrent major depressive disorder in partial remission co-occurrent with anxiety)  35615154 (Recurrent major depressive disorder co-occurrent with anxiety in full remission)  35615153 (Recurrent moderate major depressive disorder co-occurrent with anxiety)  35615152 (Recurrent severe major depressive disorder co-occurrent with anxiety)  35615151 (Recurrent mild major depressive disorder co-occurrent with anxiety)  35610109 (Recurrent depression with current moderate episode)  35610108 (Recurrent depression with current severe episode without psychotic features)  35610097 (Recurrent depression with current severe episode and psychotic features)  35609845 (Reactive depression, first episode)  35609844 (Reactive depression, recurrent)  35609843 (Reactive depression, single episode)  35609842 (Prolonged single episode of reactive depression)  35609824 (Recurrent reactive depressive episodes, severe, with psychosis)  4338031 (Mixed anxiety and depressive disorder)  4338029 (Masked depression)  4336980 (Generalized neuromuscular exhaustion syndrome)  4336957 (Mild major depression)  4333687 (Depressive conduct disorder)  4333679 (Endogenous depression first episode)  4332994 (Post-schizophrenic depression)  4327337 (Severe major depression without psychotic features)  4324959 (Recurrent major depressive disorder with postpartum onset)  4323418 (Major depression single episode, in partial remission)  4314692 (Reactive depression)  4308866 (Agitated depression)  4307951 (Primary dysthymia)  4307111 (Moderate major depression)  4305966 (Postoperative depression)  4304140 (Recurrent major depressive disorder with atypical features)  4299785 (Severe major depression, single episode, with psychotic features, mood-congruent)  4287238 (Major depressive disorder, single episode with catatonic features)  4282316 (Recurrent major depression)  4282096 (Major depression, single episode)  4270907 (Major depressive disorder, single episode with melancholic features)  4269493 (Major depression in full remission)  4263770 (Secondary dysthymia late onset)  4263748 (Recurrent major depression in full remission)  4250023 (Severe major depression with psychotic features)  4243822 (Severe major depression with psychotic features, mood-incongruent)  4243308 (Primary dysthymia early onset)  4242733 (Premenstrual dysphoric disorder)  4239471 (Postpartum depression)  4228802 (Mild recurrent major depression)  4226155 (Recurrent brief depressive disorder)  4224940 (Schizoaffective disorder, depressive type)  4224639 (Secondary dysthymia)  4223090 (Menopausal depression)  4220023 (Recurrent major depressive disorder with catatonic features)  4205471 (Recurrent major depressive disorder with melancholic features)  4197222 (Stuporous depression)  4195680 (Primary dysthymia late onset)  4195572 (Mild major depression, single episode)  4181807 (Major depressive disorder, single episode with atypical features)  4176002 (Major depression in remission)  4175329 (Organic mood disorder of depressed type)  4174987 (Minor depressive disorder)  4168858 (Endogenous depression - recurrent)  4154805 (Involutional depression)  4154391 (Major depression, melancholic type)  4154309 (Severe recurrent major depression with psychotic features)  4152280 (Major depressive disorder)  4151170 (Moderate depression)  4150047 (Secondary dysthymia early onset)  4149321 (Severe depression)  4149320 (Mild depression)  4148630 (Major depression in partial remission)  4145216 (Premenstrual dysphoric disorder in remission)  4144233 (Severe major depression with psychotic features, mood-congruent)  4141454 (Recurrent major depression in partial remission)  4141292 (Severe recurrent major depression with psychotic features, mood-congruent)  4133073 (Maternity blues)  4129842 (Mild postnatal depression)  4129184 (Severe postnatal depression)  4114950 (Endogenous depression)  4103574 (Chronic depression)  4103126 (Drug-induced depressive state)  4102973 (Postviral depression)  4098302 (Recurrent depression)  4096229 (Early onset dysthymia)  4094358 (Chronic recurrent major depressive disorder)  4093584 (Major depressive disorder, single episode with postpartum onset)  4092239 (Seasonal affective disorder)  4077577 (Moderate recurrent major depression)  4067409 (Severe major depression, single episode, with psychotic features, mood-incongruent)  4057218 (Late onset dysthymia)  4049623 (Moderate major depression, single episode)  4038252 (O/E - depressed)  4034842 (Severe recurrent major depression with psychotic features, mood-incongruent)  4031328 (Chronic major depressive disorder, single episode)  4025677 (Single episode of major depression in full remission)  442306 (Adjustment disorder with depressed mood)  441534 (Severe major depression, single episode, without psychotic features)  440698 (Brief depressive adjustment reaction)  440383 (Depressive disorder)  439259 (Single major depressive episode, severe, with psychosis)  438998 (Recurrent major depressive episodes, mild)  438727 (Atypical depressive disorder)  438406 (Severe major depression, single episode, with psychotic features)  435520 (Reactive depressive psychosis)  435220 (Severe recurrent major depression without psychotic features)  434911 (Recurrent major depressive episodes, severe, with psychosis)  433991 (Recurrent major depression in remission)  433751 (Prolonged depressive adjustment reaction)  433440 (Dysthymia)  432883 (Recurrent major depressive episodes, moderate)  432285 (Recurrent major depressive episodes) |
| Anxiety disorder | SNOMED | 42538739 (Obsessive compulsive disorder caused by synthetic cathinone)  42538738 (Anxiety disorder caused by synthetic cathinone)  42538593 (Obsessive compulsive disorder caused by stimulant)  42538592 (Anxiety disorder caused by stimulant)  42537777 (Anxiety disorder caused by synthetic cannabinoid)  37309777 (Anxiety disorder caused by methamphetamine)  37119146 (Anxiety disorder caused by opioid)  37110473 (Obsessive compulsive disorder caused by psychoactive substance)  37110466 (Anxiety disorder caused by ketamine)  37110465 (Anxiety disorder caused by dissociative drug)  37110453 (Anxiety disorder caused by methylenedioxymethamphetamine)  37110440 (Obsessive compulsive disorder caused by cocaine)  37109206 (Anxiety disorder caused by drug)  4338031 (Mixed anxiety and depressive disorder)  4277739 (Panic disorder without agoraphobia with moderate panic attacks)  4221253 (Panic disorder without agoraphobia with panic attacks in full remission)  4219044 (Amphetamine-induced anxiety disorder)  4218624 (Panic disorder without agoraphobia with mild panic attacks)  4211231 (Panic disorder without agoraphobia)  4206779 (Phencyclidine-induced anxiety disorder)  4205119 (Panic disorder without agoraphobia with panic attacks in partial remission)  4199892 (Anxiety disorder due to a general medical condition)  4198826 (Cocaine-induced anxiety disorder)  4182119 (Panic disorder without agoraphobia with severe panic attacks)  4178114 (Psychoactive substance-induced organic anxiety disorder)  4146660 (Alcohol-induced anxiety disorder)  4099956 (Acute panic state due to acute stress reaction)  4056690 (Sedative, hypnotic AND/OR anxiolytic-induced anxiety disorder)  442077 (Anxiety disorder)  436074 (Panic disorder)  434613 (Generalized anxiety disorder)  381537 (Organic anxiety disorder) |
| Conduct disorder/Oppositional defiant disorder | SNOMED | 44782933 (Conduct disorder in remission)  42538606 (Childhood onset conduct-dissocial disorder)  37110484 (Adolescent onset conduct-dissocial disorder with normal prosocial emotions)  37110483 (Adolescent onset conduct-dissocial disorder with limited prosocial emotions)  37110482 (Adolescent onset conduct-dissocial disorder)  37110481 (Childhood onset conduct-dissocial disorder with normal prosocial emotions)  37110480 (Childhood onset conduct-dissocial disorder with limited prosocial emotions)  37110479 (Oppositional defiant disorder without chronic irritability-anger with normal prosocial emotions)  37110478 (Oppositional defiant disorder without chronic irritability-anger with limited prosocial emotions)  37110477 (Oppositional defiant disorder without chronic irritability-anger)  37110476 (Oppositional defiant disorder co-occurrent with chronic irritability-anger with normal prosocial emotions)  37110475 (Oppositional defiant disorder co-occurrent with chronic irritability-anger)  4338038 (Conduct disorder - in family context)  4335176 (Conduct disorder - unsocialized)  4333687 (Depressive conduct disorder)  4279455 (Conduct disorder, group type)  4268025 (Conduct disorder, undifferentiated type)  4254395 (Conduct disorder, solitary aggressive type)  4146721 (Unsocial childhood truancy)  4105183 (Childhood disorder of conduct and emotion)  4100089 (Sibling jealousy)  4099966 (Neurotic delinquency)  4099964 (Group delinquency)  443617 (Conduct disorder)  441547 (Oppositional defiant disorder)  440697 (Nonaggressive unsocial conduct disorder)  439800 (Conduct disorder, adolescent-onset type)  437843 (Conduct disorder, childhood-onset type)  433451 (Aggressive unsocial conduct disorder)  432877 (Socialized behavior disorder) |
| Psychosis | SNOMED | 435783 (Schizophrenia),  433450 (Paranoid schizophrenia),  4008566 (Undifferentiated schizophrenia),  4101149 (Non-organic psychosis),  4335169 (Acute transient psychotic disorder),  4286201 (Schizoaffective disorder),  439004 (Residual schizophrenia),  4153292 (Schizoaffective disorder, manic type),  35622934 (Psychosis and severe depression co-occurrent and due to bipolar affective disorder),  4133495 (Acute schizophrenia-like psychotic disorder),  433996 (Catatonic schizophrenia),  441828 (Disorganized schizophrenia),  4100247 (Organic psychotic condition),  4152971 (Schizoaffective disorder, mixed type),  37016741 (Acute polymorphic psychotic disorder co-occurrent with symptoms of schizophrenia),  436067 (Simple schizophrenia),  4224940 (Schizoaffective disorder, depressive type),  434900 (Drug-induced psychosis),  37016719 (Acute polymorphic psychotic disorder without symptoms of schizophrenia),  436952 (Induced psychotic disorder),  42539146 (Psychotic disorder caused by stimulant),  37110448 (Psychotic disorder caused by volatile inhalant) |
| Sleep disorder | SNOMED | 4102985 (Nonorganic insomnia),  439708 (Disorders of initiating and maintaining sleep),  435524 (Sleep disorder),  442588 (Obstructive sleep apnea syndrome),  374905 (Non-organic sleep disorder),  313459 (Sleep apnea),  4047912 (Hypersomnia of non-organic origin),  437854 (Cataplexy and narcolepsy),  4232324 (Sleep terror disorder),  4108537 (Disorders of excessive somnolence),  377535 (Sleep walking disorder),  377266 (Non-organic disorder of the sleep-wake schedule),  439794 (Central sleep apnea syndrome),  435786 (Disorder of sleep-wake cycle),  4262580 (Primary sleep apnea of newborn) |
| Tic disorder | SNOMED | 37312584 (Transient motor tic),  37119137 (Infection causing tic),  37110326 (Tic due to and following infection),  36716895 (Primary tic disorder),  36716791 (Tic due to developmental disorder),  36716790 (Secondary tic disorder),  4299573 (Habit tic affecting hair),  4299571 (Habit tic),  4296290 (Atypical tic disorder),  4291439 (Habit tic affecting skin),  4250625 (Chronic vocal tic disorder),  4226426 (Transient tic disorder, single episode),  4210636 (Transient tic disorder),  4196373 (Recurrent transient tic disorder),  4048017 (Cluster tic syndrome),  4047760 (Facial tic disorder),  4046101 (Gestural tic disorder),  4044062 (Motor tic disorder),  4044061 (Drug-induced tic),  4043396 (Vocal tic disorder),  4034990 (Dysphonia of Gilles de la Tourette's syndrome),  762975 (Acute tic disorder),  381839 (Tic disorder),  379782 (Gilles de la Tourette's syndrome),  377265 (Transient childhood tic),  375221 (Chronic motor tic disorder) |
| Methylphenidate | RxNorm | 705944 (methylphenidate),  42943598 (Methylphenidate 10 MG Oral Tablet [PENID]),  42943576 (Methylphenidate 18 MG Extended Release Oral Tablet [CONCERTA OROS]),  42943592 (Methylphenidate 27 MG Extended Release Oral Tablet [CONCERTA OROS]),  42943580 (Methylphenidate 20 MG Extended Release Oral Capsule [Metadate CD]),  42943600 (Methylphenidate 10 MG Extended Release Oral Capsule [Metadate CD]),  42943588 (Methylphenidate 36 MG Extended Release Oral Tablet [CONCERTA OROS]),  42943584 (Methylphenidate 30 MG Extended Release Oral Capsule [Metadate CD]),  42943578 (Methylphenidate 54 MG Extended Release Oral Tablet [CONCERTA OROS]),  40236419 (methylphenidate hydrochloride 10 MG Oral Tablet),  21119719 (Methylphenidate 20 MG Extended Release Oral Capsule [Medikinet]),  40843492 (Methylphenidate 10 MG Extended Release Oral Capsule [Medikinet]),  42943601 (Methylphenidate 10 MG Extended Release Oral Capsule [BISPHENTIN]),  21080489 (Methylphenidate 30 MG Extended Release Oral Capsule [Medikinet]),  42943585 (Methylphenidate 30 MG Extended Release Oral Capsule [BISPHENTIN]),  40843487 (Methylphenidate 5 MG Extended Release Oral Capsule [Medikinet]),  42943590 (Methylphenidate 60 MG Extended Release Oral Capsule [BISPHENTIN]), |
| Anti-ADHD drugs | RxNorm | 40222068 (bupropion hydrochloride 150 MG Extended Release Oral Tablet [Wellbutrin]),  44048315 (Bupropion 300 MG Extended Release Oral Tablet [Wellbutrin XL]),  750982 (bupropion),  42707371 (clonidine hydrochloride 0.15 MG Oral Tablet),  40227886 (clonidine hydrochloride 0.1 MG Extended Release Oral Tablet [Kapvay]),  19098491 (atomoxetine 40 MG Oral Capsule [Strattera]),  742228 (atomoxetine 25 MG Oral Capsule [Strattera]),  44125936 (Bupropion 150 MG Extended Release Oral Tablet [Wellbutrin XL]),  742185 (atomoxetine),  742230 (atomoxetine 60 MG Oral Capsule [Strattera]),  1398937 (clonidine),  742227 (atomoxetine 18 MG Oral Capsule [Strattera]),  742226 (atomoxetine 10 MG Oral Capsule [Strattera]),  40169601 (clonidine hydrochloride 0.15 MG/ML Injectable Solution),  742266 (atomoxetine 80 MG Oral Capsule [Strattera]) |

# Table S2. Baseline characteristics of the study population investigating conduct disorder and ODD in the primary analysis

|  | Before matching | | | After matching | | |
| --- | --- | --- | --- | --- | --- | --- |
|  | Long-term users  (n = 1,611) | Short-term users  (n = 3,415) | Absolute SMD^*^ | Long-term users  (n = 1605) | Short-term users  (n = 2,806) | Absolute SMD^*^ |
| Age, mean (SD) | 9.0 $\pm$ 2.8 | 9.2 $\pm$ 3.0 | 0.08 | 8.9 $\pm$ 2.8 | 8.9 $\pm$ 2.7 | 0.01 |
| Gender, male | 1,339 (83.1) | 2,717 (79.6) | 0.09 | 1,237 (83.2) | 2,072 (82.1) | 0.01 |
| Neuropsychiatric disease history, n (%) |  |  |  |  |  |  |
| Anxiety disorder | 244 (15.1) | 421 (12.3) | 0.17 | 189 (12.7) | 336 (13.3) | 0.02 |
| Bipolar disorder | 172 (10.7) | 218 (6.4) | 0.17 | 130 (8.8) | 185 (7.3) | 0.03 |
| Autism spectrum disorder | 144 (8.9) | 233 (6.8) | 0.16 | 108 (7.3) | 203 (8.0) | 0.04 |
| Sleep disorder | 30 (1.9) | 61 (1.8) | 0.03 | 26 (1.8) | 39 (1.5) | 0.03 |
| Tic disorder | 189 (11.7) | 252 (7.4) | 0.16 | 150 (10.1) | 219 (8.7) | 0.04 |
| Depressive disorder | 439 (27.3) | 805 (23.6) | 0.26 | 345 (23.3) | 669 (26.5) | 0.05 |
| Psychotic disorder | 86 (5.3) | 87 (2.5) | 0.10 | 67 (4.5) | 84 (3.3) | 0.03 |
| Daily methylphenidate dose, mean (SD) | 22.8 $\pm$ 8.9 | 19.7 $\pm$ 8.7 | 0.35 | 22.2 $\pm$ 8.4 | 21.4 $\pm$ 8.6 | 0.03 |

^*^SMD: Standardized mean difference, SD: Standard deviation

# Table S3. Baseline characteristics of the study population investigating psychotic disorder in the primary analysis

|  | Before matching | | | After matching | | |
| --- | --- | --- | --- | --- | --- | --- |
|  | Long-term users  (n = 1,863) | Short-term users  (n = 3,833) | Absolute SMD^*^ | Long-term users  (n = 1,853) | Short-term users  (n = 3,181) | Absolute SMD^*^ |
| Age, mean (SD) | 9.0 $\pm$ 2.8 | 9.2 $\pm$ 2.9 | 0.07 | 9.0 $\pm$ 2.7 | 8.9 $\pm$ 2.7 | 0.01 |
| Gender, male | 1,559 (83.7) | 3,072 (80.1) | 0.10 | 1,435 (83.6) | 2,362 (83.2) | 0.01 |
| Neuropsychiatric disease history, n (%) |  |  |  |  |  |  |
| Anxiety disorder | 286 (15.4) | 469 (12.2) | 0.18 | 237 (13.8) | 369 (13.0) | 0.06 |
| Bipolar disorder | 198 (10.6) | 245 (6.4) | 0.17 | 154 (9.0) | 212 (7.5) | 0.04 |
| Autism spectrum disorder | 154 (8.3) | 244 (6.4) | 0.15 | 119 (6.9) | 222 (7.8) | 0.03 |
| Sleep disorder | 33 (1.8) | 62 (1.6) | 0.02 | 28 (1.6) | 39 (1.4) | 0.03 |
| Tic disorder | 226 (12.1) | 293 (7.6) | 0.15 | 186 (10.8) | 265 (9.3) | 0.02 |
| Depressive disorder | 522 (28.0) | 910 (23.7) | 0.27 | 414 (24.1) | 735 (25.9) | 0.02 |
| Conduct disorder and ODD | 237 (12.7) | 373 (9.7) | 0.17 | 206 (12.0) | 319 (11.2) | 0.05 |
| Daily methylphenidate dose, mean (SD) | 23.1 $\pm$ 9.0 | 19.7 $\pm$ 8.8 | 0.38 | 22.4 $\pm$ 8.5 | 21.4 $\pm$ 8.6 | 0.07 |

^*^SMD: Standardized mean difference, SD: Standard deviation

# Table S4. Comparison between methylphenidate long-term and short-term users among ADHD patients using different treatment periods

| The cutoff value for treatment periods | Outcome of interest | Number of patients | | Number of outcome | | HR [95% CI] | P-value |
| --- | --- | --- | --- | --- | --- | --- | --- |
|  |  | Long-term users | Short-term users | Long-term users | Short-term users |  |  |
| 9-month | Depression | 1,140 | 1,764 | 72 | 192 | 0.56 [0.43–0.74] | < .001 |
|  | Conduct disorder/ODD | 1,271 | 2,040 | 50 | 121 | 0.66 [0.47–0.91] | .012 |
|  | Psychotic disorder | 1,462 | 2,285 | 13 | 34 | 0.60 [0.31–1.13] | .112 |
| 15- month | Depression | 1,240 | 2,317 | 94 | 220 | 0.73 [0.57–0.93] | .010 |
|  | Conduct disorder/ODD | 1,525 | 2,649 | 44 | 137 | 0.55 [0.39–0.77] | < .001 |
|  | Psychotic disorder | 1,786 | 3,019 | 27 | 48 | 0.95 [0.59–1.52] | .829 |

# Table S5. Comparison between methylphenidate long-term and short-term users among ADHD patients who never exposed to other anti-ADHD medications

| Outcome of interest | Number of patients | | Number of outcome | | HR [95% CI] | P-value |
| --- | --- | --- | --- | --- | --- | --- |
|  | Long-term users | Short-term users | Long-term users | Short-term users |  |  |
| Depression | 1,009 | 1,702 | 62 | 155 | 0.69 [0.52–0.91] | .009 |
| Conduct disorder/ODD | 1,116 | 1,933 | 25 | 99 | 0.43 [0.28–0.67] | < .001 |
| Psychotic disorder | 1,279 | 2,138 | 24 | 14 | 1.21 [0.65–2.25] | .548 |

# Table S6. Comparison between methylphenidate non-users and methylphenidate long-term/short-term users

| Target MPH users | Outcome of interest | Number of patients | | Number of outcome | | HR [95% CI] | P-value |
| --- | --- | --- | --- | --- | --- | --- | --- |
|  |  | Target MPH user | MPH non-user | Target MPH user | MPH non-user |  |  |
| Long-term | Depression | 1,387 | 2,746 | 100 | 330 | 0.59 [0.47–0.74] | < .001 |
|  | Conduct disorder/ODD | 1,589 | 3,153 | 54 | 182 | 0.58 [0.43–0.79] | < .001 |
|  | Psychotic disorder | 1,849 | 3,649 | 31 | 75 | 0.82 [0.54–1.24] | .348 |
| Short-term | Depression | 2,763 | 5,521 | 300 | 672 | 0.91 [0.79–1.04] | .157 |
|  | Conduct disorder/ODD | 3,410 | 6,817 | 198 | 369 | 1.09 [0.91–1.29] | .349 |
|  | Psychotic disorder | 3,828 | 7,644 | 64 | 145 | 0.88 [0.66–1.19] | .406 |
